# Knowledge and Attitudes Regarding Organ Transplantation Among Cyprus Residents

**DOI:** 10.1097/jnr.0000000000000409

**Published:** 2020-11-05

**Authors:** Evanthia ASIMAKOPOULOU, Vaso STYLIANOU, Ioannis DIMITRAKOPOULOS, Alexandros ARGYRIADIS, Panagiota BELLOU–MYLONA

**Affiliations:** 1 PhD, RN, Lecturer, School of Health Sciences, Frederick University, Nicosia, Cyprus; 2 PhD(c), RN, Staff Nurse, School of Health Sciences, Frederick University, Nicosia, Cyprus; 3 MSc, RN, Special Teaching Staff, School of Health Sciences, Frederick University, Nicosia, Cyprus; 4 PhD, RN, Assistant Professor, School of Health Sciences and School of Education and Social Sciences, Frederick University, Nicosia, Cyprus; 5 PhD, Professor, School of Health Sciences, Frederick University, Nicosia, Cyprus.

**Keywords:** attitudes, Cyprus, knowledge levels, organ donation, transplantation

## Abstract

**Background:**

Organ transplantation was one of the greatest achievements of medical science during the 20th century. Knowledge, education, and culture all play prominent roles in transplantation because of the complexity of the process from donation to transplantation.

**Purpose:**

The aim of this research was to determine and analyze the knowledge and attitudes about organ donation and transplantation among the general population in Limassol, Cyprus.

**Methods:**

A quantitative research approach was followed, and a questionnaire consisting of closed-ended questions was completed by adults from the general population in Limassol.

**Results:**

One thousand two hundred adults out of the 1,346 adults who were contacted responded to the survey (response rate: 89%) and were included as participants. Of the participants, 93.4% (*p* < .05) considered organ donation to be lifesaving, 57% expressed interest (and 39.8% expressed disinterest) in becoming organ donors, 80.6% (*p* < .05) expressed awareness of there being a waiting list for people in need of organ transplantation, 50.4% agreed that brain death must be confirmed before organ removal for transplantation, and 47% recalled having been informed about organ donation through the media, with 31.5% stating that they had never been informed about organ donation.

**Conclusions:**

The participants demonstrated limited awareness regarding the organ donation system in Cyprus. Furthermore, a significant percentage stated that they lacked a source for obtaining related information. The Cypriot society should be informed and encouraged to participate in organ donation to increase the rate of organ transplantation.

## Introduction

Transplantation, one of the most progressive areas in the healthcare sciences, has moved from experimental trials to acceptable therapies and is being used to treat many severe health problems (Shafran et al., 2014). Organ donation is a voluntary process that leads to the transfer of life to a person who needs a healthy organ to live or to improve their quality of life (Chen et al., 2007). This action has no financial benefit and thus represents a purely a humanitarian effort (Voo, 2015).

According to Caplan (2009), about a dozen people in the United States waiting for organ transplants die each day. Many deaths may be prevented if more organs are available. This pressure is getting worse because waiting lists are growing faster than the supply of organs. Therefore, healthcare professionals must consider new options for including more people in organ donation programs, and transplantation centers may need to reconsider their waiting list criteria and priorities (Caplan, 2009; Shafran et al., 2014). The decisions related to organ transplantation involve a myriad of difficult ethical and legal issues.

The first organ transplant attempts in Cyprus were made in 1986 from living kidney donors. Since then, only kidney transplants have been performed in Cyprus, while patients who need other organs have been required to list abroad (Kyriakides et al., 2002). There is an intensive need to expand the transplant methods used in Cyprus and to include many other organs in the process. Moreover, there is a lack of transplant coordinators and education for healthcare professionals in Cyprus.

Transplantation, which is the implantation of a tissue, cell, or organ (called a graft), is often the only effective treatment for organ failure (Cho et al., 2018). The process of transplanting organs from donors to patients is governed by strict rules and regulations (Prabhu, 2019). A transplant from a living donor has many advantages, as it is a planned operation in which the person is hemodynamically stable and well oxygenated (Tong et al., 2013). However, donors who have experienced brain death and are hospitalized in intensive care units are the primary source of transplants, which requires tests and checks by doctors in different specialties at different times to confirm the time of brain death, qualify the donation, and confirm compatibility (Chinen & Buckley, 2010). The total process is very complicated and time sensitive. Transport and storage conditions for donated organs must follow applicable European directives. In addition, European Union member states must ensure that transplantation takes place in or under the supervision of transplantation centers that comply with the rules of the European Union (Bouwman et al., 2013).

Social media networks are an important factor driving changing views and attitudes toward organ transplantations. Social networks may positively affect public opinion through information campaigns about organ donation (Cameron et al., 2013; Jiang et al., 2019). On the other hand, misinformation about transplants, which confuses public opinion and leads to distrust in the medical community, is considered an underlying cause of low organ donor registration rates (Cameron et al., 2013). Religion is also an important factor that influences personal decision-making regarding becoming an organ donor. If a patient has an irreversible medical condition and observes all legal protocols, churches are generally in favor of organ donation (Bruzzone, 2008).

Donation decisions are influenced by religious, cultural, altruistic, and knowledge-based beliefs (Ríos et al., 2018). In addition, family, school, and the state affect related directions and policies on this issue. School courses, government campaigns, and social media have all been used to promote organ donation awareness and registration.

Because of the intense interest in transplantation and of Cyprus’ high position on the European transplantation charts (Kyriakides et al., 2002) in terms of living donors, this study was conducted to assess the attitudes and knowledge of Limassol residents about organ transplantation.

## Methods

The survey, conducted in Limassol, Cyprus, adopted a quantitative approach because of the main aim of this study. The research tool used was the questionnaire, and the distribution of the questionnaires took place in public areas where people were waiting to access public services. The sample used in the analysis included 1,200 adult men and women of all education levels. Data collection lasted 4 months, from June 2017 to September 2017.

The sampling method used was convenience sampling. Despite the risks, this method is very useful for investigating phenomena because of its low cost, deep access to information, and the relatively brief time required to collect data (Gravetter & Forzano, 2011).

The items on the questionnaire were drafted after a thorough literature review and were checked by two relevant experts for appropriateness (Bedi et al., 2015; Burra et al., 2005; Tsavdaroglou et al., 2013). The questionnaire was then pilot tested on a sample of 10 individuals for ease of comprehension and item appropriateness. These 10 individuals were not included in the main study. The original questionnaire consisted of a smaller number of questions. After the pilot study, adjustments were made to address several difficulties noted regarding terminology. The questionnaire was divided into four sections: sociodemographic details, awareness regarding donation, experience with organ donation, and intentions regarding donation. Each participant was given 10 minutes to complete the questionnaire, and informed consent was obtained from all participants beforehand.

Each correct answer was assigned a score of 1, and each wrong or equivocal (i.e., I do not know) answer was assigned a score of 0. Next, a total score for each participant was calculated, divided by the total number of questions (*n* = 12), and then multiplied by 100 to extract a percentage scale (0–100). A larger percentage scale value indicated better knowledge, with a minimum scale value of 60% used to distinguish between “inadequate” and “adequate” knowledge categories, having responded correctly to most of the questions (at least 6 out of 10; Albert et al., 2002; Sánchez-Mendiola et al., 2012; Testa et al., 2018).

Data analysis for this study was conducted using IBM SPSS Version 21.0 (IBM, Inc., Armonk, NY, USA). The reliability coefficients and Cronbach’s internal consistency of the attitudes scale were assessed. The knowledge score, frequency distributions of the participants’ characteristics, estimated relative frequencies, and 95.0% confidence intervals were calculated, and a chi-square test was used in categorical data. Differences in knowledge scale attributable to sociodemographic characteristics and heterogeneity were tested using the variance analysis method and the Levene method, respectively. The difference between high and low knowledge scale attributable to sociodemographic characteristics was checked by a chi-square test, and the acceptable level of significance was set at .05 (Linardakis & Dellaportas, 2003).

The National Bioethics Committee of Cyprus and the Data Protection Office approved the study (Protocol No. ΕΕΒΚ ΕΠ2014.01.105).

## Results

Less than two thirds (59.7%; highest notable frequency, *p* < .05) responded correctly to the questionnaire statement that someone must be completely healthy to be an organ donor, 80.6% responded that there is a waiting list for recipients, 50.4% responded that brain death must be confirmed before organ removal, 67.2% (highest notable frequency, *p* < .05) responded that all donors are tested for transmittable diseases, 69.1% responded that you may change your mind after registering as a donor, 45.5% responded that anyone may become a donor regardless of their age, 42.3% responded that individuals may designate who their organs will be given to in case of brain death, and 93.4% (highest notable frequency, *p* < .05) responded that organ donation saves lives. Less than half (45.1%; highest notable frequency, *p* < .05) responded that they were not aware of the view of the church toward organ donation; 19.2% (lowest notable frequency, *p* < .05) did not know whether, in case of brain death, the next of kin could make the decision regarding donation; and 27.8% (lowest notable frequency, *p* < .05) did not know if brain death was reversible (Table 1).

**Table 1.** Awareness Score According to the Correct Answers Given in the 12 Questions Regarding Organ Donation in Correspondence to the Demographic Details of the Participants

Characteristic	Awareness Score					*p*
	*n*	*M*	*SD*	*p*	Score ≥ 60 (%)	
Total	1,200	52.3	17.7	—	28.5	—
Gender				.876		.053
Male	519	52.4	19.1		31.4	
Female	681	52.3	16.5		26.3	
Age (years)				.040*		.014*
18–29	392	51.3	17.7		26.3	
30–39	403	51.4	16.9		25.3	
40–49	196	54.5	17.9		34.7	
50–59	156	53.2	18.9		31.4	
≥ 60	53	56.0	17.9		37.7	
Education				.280		.461
Up to primary school	25	54.3	23.3		32.0	
Secondary school	45	55.7	18.6		35.6	
High school	353	51.6	17.2		25.5	
Technological institute	148	50.0	18.9		25.7	
University	629	52.9	17.3		30.2	

*p < .05.

The highest percentage of participants who had been informed about organ donation by a doctor were those with a primary level of education (24%), whereas only 9.1% of those had university education. A large percentage of participants at all levels of education indicated having never received information on organ donation, with the highest percentages being 35.4% and 30.7%, respectively, for participants educated to the high school and university levels (Table 2).

**Table 2.** Frequencies of Stated Sources of Information Regarding Organ Donation, by Level of Education

Source of Information^a^	Education Level (%)					*p*
	Up to Primary School	Secondary School	High School	Tech. Institute	University	
Doctor	24.0	20.0	10.5	15.5	9.1	.015*
Family	16.0	11.1	17.6	17.3	13.5	.131
Close contacts	32.0	26.7	30.0	30.4	31.2	.638
Mass media	60.0	44.4	45.6	43.2	48.5	.716
Medical journals	20.0	17.8	15.0	12.8	15.1	.670
Conventions—events	4.0	4.4	2.0	4.7	3.3	.592
University	0.0	0.0	0.0	2.0	1.0	.107
Movies	0.0	0.0	0.3	0.0	0.8	.188
No one (no source)	24.0	28.9	35.4	27.7	30.7	.525

^a^Multiple choice.

*p < .05.

Over half, 61.8% and 52.9% (highest notable frequency, *p* < .05), respectively, responded negatively about knowing a family member or a close acquaintance needing a transplant or previously declaring an interest in becoming a donor and whether the people in their environment held negative attitudes toward donation. A high percentage (50.8%; highest notable frequency, *p* < .05) responded that they did not know if anyone in their family or close environment had shown interest in becoming an organ donor, whereas 57.0% stated that they would become a donor and 39.8% responded that they would not become a donor (highest notable frequency, *p* < .05). Slightly over half (52.3%; highest notable frequency, *p* < .05) agreed strongly that the reason they would like to become a donor is because they desired to help their fellow man. Respective percentages of 39.5%, 34.5%, and 44.7% (highest notable frequency, *p* < .05) completely disagreed, which could be affected or sensitized by a family member or a friend who was either a donor or needed a transplant and become a donor only for someone dear to them.

Respective percentages of 40.3%, 41.1%, and 37.7% (highest notable frequency, *p* < .05) answered affirmatively to being afraid, to not trusting the unions and the doctors about how they will be treated knowing that they are donors, and to being unsure whether their transplanted organs would be used properly. Respective percentages of 37.1% and 44.9% (highest notable frequency, *p* < .05) agreed about not being interested in organ donation and on not being sufficiently aware of the issue of organ donation. More than 4 of 10 participants (42.1%; highest notable frequency, *p* < .05) questioned brain death and agreed that there was still hope for life until the last breath. However, 44.2% (highest notable frequency, *p* < .05) accepted brain death as physical death (Table 3). Respective percentages of 32.0%, 37.3%, and 32.7% (highest notable frequency, *p* < .05) agreed that they were positively affected by mass media, the title of organ donor gives a sense of satisfaction and pride, and organ donation offers a way to live on after death (Table 3).

**Table 3.** Agreeability Degree Frequencies From the Participants Who Did Not Express/Expressed Willingness to Become a Donor (*y* = 474) in Questions About Personal Views Regarding Organ Donation

Question	Completely Disagree	Disagree	Agree	Completely Agree	I Do Not Know
	%	%	%	%	%
Unwilling					
1. I’m afraid.	13.6	23.9	40.3*	13.8	8.4
2. I do not trust the unions to provide needed treatments to registered donors.	8.8	24.5	41.1*	15.9	9.6
3. I do not trust doctors and the way that I would be treated during hospitalization as a registered donor.	11.1	27.3	37.9*	16.8	6.9
4. I do not believe that the transplant would be used correctly.	11.7	29.6	37.7*	14.3	6.7
5. I find it irrelevant and am not really concerned about the matter.	26.8	44.2*	13.4	5.9	9.6
6. I disregard the matter and have not given it any serious thought.	15.3	24.9	37.1*	18.0	4.6
7. I’m still not aware or decided regarding organ donation.	13.4	19.7	44.9*	17.4	4.6
8. I question brain death, since there is still hope for life until the last breath.	7.5	20.8	42.1*	28.3	1.3
Willing					
1. I really want to help a fellow person.	2.3	2.3	41.5	52.3*	1.5
2. By donating an organ, you are saving a life, which is something that agrees with my religious beliefs.	7.5	7.6	38.0	38.9	8.0
3. I have been affected by a family member or a friend that is a donor.	39.5*	28.9	14.6	7.7	9.2
4. I have been sensitized by a family member or friend that needed a transplant.	34.5*	24.6	19.7	13.9	7.3
5. I would only become a donor for someone dear to me.	44.7*	30.1	12.6	7.7	4.8
6. The mass media has had a positive effect on me about becoming a donor.	24.6	21.9	32.0*	12.3	9.2
7. The title of organ donor gives me a sense of satisfaction and pride.	16.5	13.9	37.3*	27.6	4.7
8. By becoming an organ donor I feel that a piece of me will live on after my death.	19.0	14.8	32.7*	25.9	7.6

*p < .05.

A higher percentage of women participants were motivated by altruistic motives than their male counterparts. Desire to limit organ donations only to close friends or family fell with rising education level (highest notable relation, *p* < .05). The percentage of those expressing willingness to become a donor and disagreeing about being sensitized by a family member or a close friend who needed a transplant rose with the education level of the participant. Women participants disagreed more than their male counterparts about being unconcerned or only mildly concerned regarding the subject of donation (highest notable relation, *p* < .05). Among those who expressed unwillingness to become a donor, older age was associated negatively with the interest in organ donation and accepting brain death as physical death (highest notable relation, *p* < .05). Among those participants who did not express willingness to become a donor, awareness level was positively associated with disagreeing about being afraid, finding the subject of organs irrelevant, and being unconcerned about organ donation.

## Discussion

The literature on the knowledge and attitudes of Cypriots regarding organ transplantation–donation is very limited. After evaluating the awareness of the participants, some important results were found in this study. A high percentage answered positively to the questions that someone must be completely healthy to become a donor and that there is a waiting list for perspective donors.

A very high percentage of the participants (93.4%) agreed that organ donation saves lives. In a similar survey in Greece (Tsavdaroglou et al., 2013), 90.5% of nursing students considered that organ donation saves lives. In a survey in Pakistan (Ali et al., 2013), 81.6% of medical students agreed that it was ethically correct to donate an organ. In another survey in Saudi Arabia (Mohamed & Guella, 2013), over 90% of a random general-population sample were aware of organ transplantation donation. Moreover, a survey conducted in an African American community in Buffalo, New York, showed high organ donation awareness, with 88% of the participants being familiar with organ donation, 36% indicating they would not donate organs, 31% indicating that they would donate, and 33% indicating being unsure (Minniefield & Muti, 2002). Similar results were also found in young British adults, who showed positive attitudes toward donation (90% are in favor of organ donation) and 63.9% and 78.2% expressing respective willingness to donate and to receive organs (Coad et al., 2013). Moreover, 72% of nonmedical hospital staffs expressed being in favor of donating organs after death in Spain and Latin American. This percentage varies greatly by country, with 98% of Cuban, 80% of Mexican, 66% of Costa Rican, and 52% of Spanish respondents responding favorably (Ríos et al., 2013).

With regard to experiences with organ donation, high percentages in this study answered that family members or close acquaintances had never needed a transplant and had never officially declared wanting to be a donor. A similar percentage expressed not knowing if any close acquaintance wanted to become a donor. However, a high percentage answered negatively to the question whether their close acquaintances held negative attitudes toward organ donation.

In this study, 57.0% agreed to become an organ donor, whereas 39.0% declined. In a similar survey in Greece (Theodorakopoulou & Bakalis, 2010), 62.0% and 68.0% of nurses and nursing students, respectively, responded they would become organ donors. In the Buffalo, New York, study (Minniefield & Muti, 2002), 33% respondents indicated that they would donate their organs, 36% said no, and 33% were unsure. In Brazil (Peron et al., 2004), 68.2% of university health students expressed willingness to be organ donors. On the other hand, more than 90% of medical students in Italy showed strongly positive attitudes; most of these were prepared to donate their organs after death, and 63% had already signed a donor card (Burra et al., 2005).

The main reasons for participants in this study not intending to be organ donors included fear (40.3%), distrust in organizations (41.1%), distrust in doctors during hospitalization (37.9%), and hope for life until their last breath (42.1%). In a survey in Greece (Theodorakopoulou & Bakalis, 2010), 32% of nurses and 17% nursing students expressed distrust in the transplantation selection process. In Saudi Arabia (Mohamed & Guella, 2013), 15.2% expressed fear of the operation, and in Brazil (Peron et al., 2004), almost 79.4% of the student participants did not believe that the waiting list for transplants was followed.

In a survey of the adult population in Pakistan regarding the allowance of organ donation by religion, “yes,” “no,” and “do not know” earned roughly equal percentages (Saleem et al., 2009). In that survey, religion was the leading reason (45.4%) expressed that organ donation should not be promoted. A meta-analysis in 2013 (Tong et al., 2013) showed that most were in favor of living-directed donation (85.5%), with the barriers identified including fear of surgery and health risks, lack of knowledge, respect for cultural norms, financial loss, distrust in hospitals, and avoiding recipient indebtedness. In addition, 32% of the sample in the survey in Buffalo, New York, stated that they did not trust doctors, and most of the young adult respondents were afraid they would not receive proper medical attention if they were registered as organ donors (Minniefield & Muti, 2002). Finally, the main reason among nursing students for hesitating to donate organs was fear of the commercialization of organ donation (Tsavdaroglou et al., 2013).

In term of religion, a high percentage in this study seemed not to know the church’s position toward donation, whereas a small percentage reflected a belief that the church forbids organ donation. This study found that a large percentage (45.1%) does not know the position of the church. From a religious point of view, 68.6% of Saudi Arabians consider it legal to donate organs and 26.2% do not (Mohamed & Guella, 2013). The African American respondents most often cited religious reasons for not donating (Minniefield & Muti, 2002).

A high percentage of participants identified the mass media as their main source of information, whereas a smaller percentage identified their close acquaintances, medical journals, and their doctor as their primary source. A significant percentage stated that they had no source of related information. This raises concerns regarding how accurately the public is being informed. In this study, 47.1% were informed by the media, 30.6% were informed by friends, and 17.6% were informed by family. Notably, a large percentage (31.5%) indicated being informed by no one. Similarly, in a study in Pakistan (Ali et al., 2013), most of the students (64.6%) were made aware of organ donation through print and electronic media, whereas only 27.8% were made aware by their healthcare providers. These results suggest that nonphysicians are frequently overlooked and are not provided with the necessary information to help them better understand the organ donation and transplantation process (Zambudio et al., 2012).

A very small percentage in this study answered that there are no regulations governing organ donation. One out of 10 (9.8%) expressed the belief that no regulations existed, whereas 45.8% were unaware whether there was related regulations in Cyprus. A study in Greece (Theodorakopoulou & Bakalis, 2010) found that 82% of nursing students and 62.5% of the nurses were unaware of related regulations. In an international study (Ramadurg & Gupta, 2014), it was found that awareness of regulations governing transplantation among medical students was poor (44.3% were unaware regarding the existence of related laws).

In cases of brain death, with regard to whether relatives could decide about organ donation regardless of the patient’s declared intent while alive, 43% of the participants in this study disagreed and 19.2% did not know. However, in another study, 61.2% of subjects expressed that relatives could donate a patient’s organs after brain death (Mohamed & Guella, 2013). Family members continue to play a prominent role in donation decisions at time of death, as many participants relied on their family to make end-of-life care decisions for them in the absence of a written advance directive (Ríos et al., 2019; Rodrigue et al., 2006).

A high percentage in this study expressed awareness that the donor must be declared brain dead in order for an organ to be removed. However, relatively high percentages of the respondents seemed not to know that brain death was irreversible, with 37.3% believing brain death to be reversible and 27.8% being unsure.

In responding to the statement that a person could predetermine where their organs would be given, 44.7% of the sample disagreed and only 12.6% agreed. In this study, a high percentage believed that anyone could donate organs no matter their age. A similar general-population survey in Pakistan (Saleem et al., 2009) found that half (51.1%) would donate their organs to a family member. In a cohort study of Latin Americans living in the United States, respondents who were in favor of donating a family member’s organs were more in favor of donating their own organs, whereas those who had previously discussed the subject of organ donation in their family circle held a more favorable attitude (Ríos et al., 2017). In a study of university students in China, 62.4% respondents indicated that designated relatives were their most probable recipients (Zhang et al., 2007).

Although this study achieved its aims, there are some limitations. The survey was conducted only in Limassol and not in the other cities of Cyprus. Therefore, to generalize the results, the study should involve participants from different areas of the country. Also, the results may have been affected by cultural bias, as the origin and cultural background of the participants were not considered.

### Conclusions

In conclusion, a high percentage of participants expressed a desire to become a donor, whereas a very small percentage expressed that they did not know. Those who expressed willingness to become an organ donor agreed that a main reason for doing so was to help their fellow man. Furthermore, high percentages of donors were found among those who were affected by mass media, those who felt the title of donor conferred a sense of satisfaction/pride, and those who felt that donating would allow a part of their being to live on after their death. A high percentage of those unwilling to become organ donors stated fear as their main reason, with distrust of doctors and concern that they would not receive necessary treatment as registered donors also noted as important reasons.

The results support that Cypriots have a relatively high level of awareness regarding organ donation. As transplant waiting lists have increased exponentially worldwide during recent decades, there is a need to develop community programs to further raise public awareness about organ and tissue donation in Cyprus. Public education, mainly through the media, nongovernmental organizations, and lectures by experts, has been the main strategy to change social attitudes toward organ donation and transplantation.
